# GWAPower: a statistical power calculation software for genome-wide association studies with quantitative traits

**DOI:** 10.1186/1471-2156-12-12

**Published:** 2011-01-21

**Authors:** Sheng Feng, Shengchu Wang, Chia-Cheng Chen, Lan Lan

**Affiliations:** 1 Deaprtment of Biostatistics and Bioinformatics, Duke University, Durham, North Carolina 27710, USA; 2 Department of Statistics, North Carolina State University, Raleigh, North Carolina 27695, USA

## Abstract

**Background:**

In designing genome-wide association (GWA) studies it is important to calculate statistical power. General statistical power calculation procedures for quantitative measures often require information concerning summary statistics of distributions such as mean and variance. However, with genetic studies, the effect size of quantitative traits is traditionally expressed as heritability, a quantity defined as the amount of phenotypic variation in the population that can be ascribed to the genetic variants among individuals. Heritability is hard to transform into summary statistics. Therefore, general power calculation procedures cannot be used directly in GWA studies. The development of appropriate statistical methods and a user-friendly software package to address this problem would be welcomed.

**Results:**

This paper presents GWAPower, a statistical software package of power calculation designed for GWA studies with quantitative traits, where genetic effect is defined as heritability. Based on several popular one-degree-of-freedom genetic models, this method avoids the need to specify the non-centrality parameter of the F-distribution under the alternative hypothesis. Therefore, it can use heritability information directly without approximation. In GWAPower, the power calculation can be easily adjusted for adding covariates and linkage disequilibrium information. An example is provided to illustrate GWAPower, followed by discussions.

**Conclusions:**

GWAPower is a user-friendly free software package for calculating statistical power based on heritability in GWA studies with quantitative traits. The software is freely available at: http://dl.dropbox.com/u/10502931/GWAPower.zip

## Background

Statistical power and sample size calculation is an important step during experiment design in genome-wide association (GWA) studies. It estimates the probability that a true genetic effect can be detected under the experimental constraints and other reasonable assumptions. To date, more than 230 GWA studies have been conducted and reported. Among these, case control design (>100 studies) and cohort studies with unrelated subjects (>70 studies) are the two most popular designs. In the majority of cohort studies, one or more phenotypic variables are quantitative traits. A number of statistical power calculation softwares have been developed for case-control GWA studies [1-5]. This report presents a simple and user-friendly statistical software, GWAPower, for power calculation of GWA studies with quantitative traits.

General statistical power calculation procedures for quantitative measures often assume normality and require information of the first two distribution moments (means and variances) of the quantitative measure. These power calculation procedures cannot be applied directly in genetic studies. One challenge is that a key parameter, i.e. the size of the hypothesized genetic effect, is represented in a genetic term, "heritability". In its broad sense, heritability is defined as the amount of phenotypic variation in the population that can be ascribed to the genetic variation among individuals. If the total variability of the phenotype is V(t) and the variations that can be explained by genetic variants and error terms are V(g) and V(e), respectively, where V(t) = V(g) + V(e), then the heritability (H) is estimated as: H = V(g)/V(t). Although H is intrinsically related to the distribution of the quantitative measure, the exact mathematical transformation formula is not clear; given the value of H, the first two moments of the quantitative measure cannot be determined directly.

However, a power calculation procedure based on heritability is possible for GWA studies, since most applied genetic models in GWA analysis are simple and have only one degree of freedom. Using straightforward algebra, a sound statistical procedure and convenient graphical user interface software have been developed to estimate the statistical power for quantitative traits in GWA studies.

## Results and Discussion

### Methods for calculating power with heritability in one-degree-of-freedom models

The heritability (H) can be estimated by the Analysis of Variance (ANOVA) approach. For independent random samples drawn from natural populations, H is the coefficient of determination, or R^2^. In general, the ANOVA F-test can be used as the base of power calculations. However, this approach requires the non-centrality parameter λ of the F-distribution, which is defined as:

$$\lambda =\sum_{i}r_{i}\tau_{i}^{2}/\sigma^{2},$$

where τ_i _is the phenotype mean of genotype group i; σ^2 ^is the error variation; and r_i _is the number of individuals in each genotype group i. Therefore, distribution parameters such as means and variances are still necessary for this approach.

We provide a solution that avoids assuming the non-centrality parameter in power calculations. In GWA data analysis, some popular genetic models have one degree of freedom including the dominant model, the recessive model and the additive model. When these genetic models are assumed, the one-way ANOVA model (dominant and recessive models) and the simple linear regression model (additive models) are used in the majority of GWA data analyses for quantitative traits. In these studies, the F-statistic is related to Student's t statistic, i.e. if a random variable X has a t distribution with *d *degrees of freedom, then X^2 ^has F distribution (1, *d*). This is true for central and non-central distributions [6]. This suggests that so long as the genetic model contains only one degree of freedom, then an F distribution can be used to perform the square root transformation to obtain the t statistics for the power calculation.

For dominant and recessive models, the one-way ANOVA table and the terminologies are described in Table 1. The heritability is defined as H = SSM/SST. Given H and the total sample size N, SSE = (1-H)SST, MSE = (1-H)SST/(N-2), and F = H(N-2)/(1-H), which is now independent of SSM and SSE. The F statistics can be converted into t statistics using the square root. The (relative) standard deviation of the t-test is std = square root (MSE). The (relative) numerator of the t-statistic is: Δm = std*t. For the additive model, assuming a linear regression model is used, the variance can be similarly decomposed. For all models, the F-statistic is a function only of H and N. The power under the two-sided t-test at level α is expressed as

**Table 1:** The ANOVA table for the dominate and the recessive model

	Degree of Freedom	Sum of Squares	Mean Squares	F
Genetic Model	P = 1	SSM	MSM=SSM	MSM/MSE
	
Error	N-p=N-2	SSE	MSE=SSE/(N-2)	
	
Total	N-1	SST=SSM+SSE

P=probability (|t|>t(N−2,α/2)).

It should be noted that (1) the absolute values of SST and SSM are unknown, as are the absolute values of numerator and denominator of the t-statistic. However, in this approach, only the relative values are necessary to calculate power; (2) the t-test is associated with the ANOVA table. In this study, an ANOVA (and the associated F-test) is used owing to its ability to accommodate the concept of heritability and to adjust for covariate effects. Therefore, if a dominant or a recessive genetic model is considered, the t-test compares two sample means within an ANOVA framework, although an ANOVA t-test is different from a "two sample t-test".

### The Software

**Figure 1:**
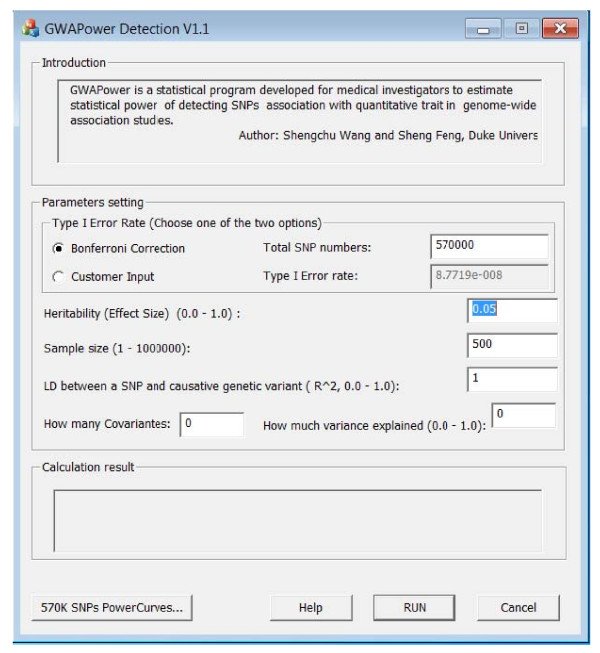
**A snapshot of the GUI interface of GWAPower**.

GWAPower calculates statistical power using the following input parameters: (1) The heritability (a number in the range 0~1); (2) Total sample size; (3) The number of SNPs in the GWA study; (4) The type 1 error rate: The default value is calculated using a Bonferroni correction. A snapshot of the main page of the software is presented in Figure 1.

GWAPower considers other design factors so that users have the options to explore the power of the study under various situations. The factors include:

(1) Covariates: users can input the percentage of the variation explained by the covariates, in addition to the genetic effects and the number of total covariates. The information is included in the ANOVA table and the statistical power is adjusted accordingly, i.e. the degree of freedom of the t-test is N-2-number of covariates;

(2) Linkage Disequilibrium (LD): users can input the two-locus LD (r^2^) between the SNP marker and the hypothesized causal variant. When the LD between the genotyped and "causative" marker is r^2^, the sample size is increased by 1/r^2^, because in order to have the same power to detect a causative variant when using a marker that is r^2 ^away, an increase in sample size of 1/r^2 ^is required [7,8].

**Figure 2:**
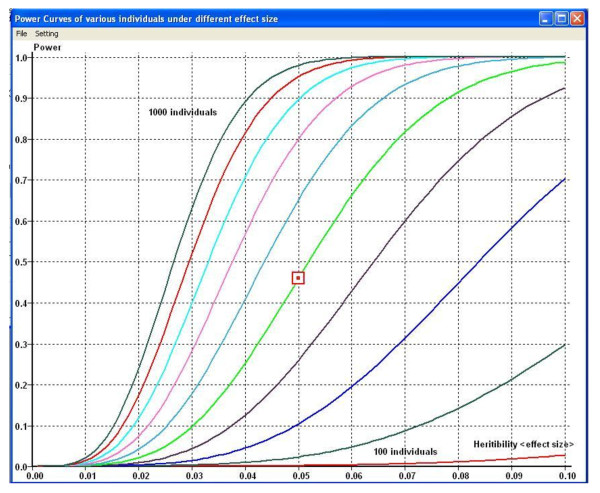
**GWAPower outputs a family of power curves**. The calculated power with parameters inputted from the main page is also identified in the graph.

The output information include: (1) the statistical power; (2) a family of power curves outputted with different heritability and sample size combinations, as demonstrated in Figure 2.

### Implementation

The GWAPower package was developed using Microsoft Visual C++ 2008 and consists a main dialog graphical user interface (GUI). Users can download and install the compiled versions of the GUI that run as stand-alone applications under Windows operating systems. A demonstration of the GUI for power calculations with the corresponding parameters setting is presented in Figure 1. By clicking the button "SNPs Power Curves", the GUI displays power curves of various sample size with H = 1% ~ 10%. The graphs can be copied into a Windows clipboard by using Menu - Copy to Clipboard. The graphs can be pasted into other Windows applications.

### Example for HIV studies

The beta-version of GWAPower has been used to support the designs of several GWA studies [9-11]. A GWA study was conducted to search for host genetic variants related to HIV set-point variation. In the first stage of the study, 486 HIV infected Caucasian subjects were genotyped, and the associations between the SNPs and one quantitative phenotype, i.e. the viral load at the set-point, were tested [10]. In the second stage, 2068 subjects were genotyped from the same population, to increase the total sample size to 2554 [11]. Using GWAPower, the statistical powers of the two stages of the studies were calculated (Table 2). The results indicated that assuming H = 7%, a sample size of 486 was adequate to provide 80% statistical power to detect the associated SNP; with the sample size increased to 2554, the smallest H that could be detected was 1.4%. In real data analyses, the largest H was estimated at 9.6%.

**Table 2:** The statistical power estimated by GWAPower for the HIV studies

Heritability	486 Subjects	2554 Subjects
1%	0.001	0.442

1.4%	0.005	**0.787**

2%	0.019	**0.977**

3%	0.088	**0.999**

4%	0.232	**0.999**

5%	0.430	**0.999**

6%	0.631	**0.999**

7%	**0.792**	**0.999**

8%	**0.897**	**0.999**

9%	**0.955**	**0.999**

10%	**0.983**	**0.999**

## Conclusions

The GWAPower package provides a simple and useful statistical power calculation procedure for GWAS with quantitative traits. It is designed specifically to allow genetic researchers to use the genetic term, heritability, instead of the general statistical term, 'phenotype means of each genotype', in power calculations. Current users have reported that the tool is very user-friendly and easy to operate.

Although GWAPower is developed for GWA studies, it can be used for general genetic studies if researchers wish to use heritability as the parameter for genetic effect size. However, this approach should be used with caution. Some complicating factors are not considered such as population stratification, various interactions among genetic variants, and environmental factors. Furthermore, Bonferroni correction for multiple tests is known to be over-conservative. Therefore, for complicated designs with multiple confounding factors, researchers are encouraged to consult a statistical geneticist.

One reviewer has reported that another software "genetic power calculator" (GPC) [1] can be used to estimate statistical power in GWA studies with quantitative traits, with some simple adjustments. A major difference between GPC and GWAPower is that GPC is based on a comprehensive mixed model approach [12], making it amenable for use in various experimental designs. GWAPower takes advantage of the fact that most GWA study designs are reasonably simple, and much simpler models such as ANOVA or regression models are often adequate. In such cases, the approximations of estimating distribution parameters are not necessary.

## Authors' contributions

SF and LL derived the method. SF and SW developed the software. SF, SW, CCC and LL wrote the manuscript. All authors read and approved the final manuscript.
